# Pepsin homologues in bacteria

**DOI:** 10.1186/1471-2164-10-437

**Published:** 2009-09-16

**Authors:** Neil D Rawlings, Alex Bateman

**Affiliations:** 1 Wellcome Trust Sanger Institute, Wellcome Trust Genome Campus, Hinxton, Cambridgeshire, CB10 1SA, UK

## Abstract

**Background:**

Peptidase family A1, to which pepsin belongs, had been assumed to be restricted to eukaryotes. The tertiary structure of pepsin shows two lobes with similar folds and it has been suggested that the gene has arisen from an ancient duplication and fusion event. The only sequence similarity between the lobes is restricted to the motif around the active site aspartate and a hydrophobic-hydrophobic-Gly motif. Together, these contribute to an essential structural feature known as a psi-loop. There is one such psi-loop in each lobe, and so each lobe presents an active Asp. The human immunodeficiency virus peptidase, retropepsin, from peptidase family A2 also has a similar fold but consists of one lobe only and has to dimerize to be active. All known members of family A1 show the bilobed structure, but it is unclear if the ancestor of family A1 was similar to an A2 peptidase, or if the ancestral retropepsin was derived from a half-pepsin gene. The presence of a pepsin homologue in a prokaryote might give insights into the evolution of the pepsin family.

**Results:**

Homologues of the aspartic peptidase pepsin have been found in the completed genomic sequences from seven species of bacteria. The bacterial homologues, unlike those from eukaryotes, do not possess signal peptides, and would therefore be intracellular acting at neutral pH. The bacterial homologues have Thr218 replaced by Asp, a change which in renin has been shown to confer activity at neutral pH. No pepsin homologues could be detected in any archaean genome.

**Conclusion:**

The peptidase family A1 is found in some species of bacteria as well as eukaryotes. The bacterial homologues fall into two groups, one from oceanic bacteria and one from plant symbionts. The bacterial homologues are all predicted to be intracellular proteins, unlike the eukaryotic enzymes. The bacterial homologues are bilobed like pepsin, implying that if no horizontal gene transfer has occurred the duplication and fusion event might be very ancient indeed, preceding the divergence of bacteria and eukaryotes. It is unclear whether all the bacterial homologues are derived from horizontal gene transfer, but those from the plant symbionts probably are. The homologues from oceanic bacteria are most closely related to memapsins (or BACE-1 and BACE-2), but are so divergent that they are close to the root of the phylogenetic tree and to the division of the A1 family into two subfamilies.

## Background

Peptidases are widespread enzymes that catalyse the hydrolysis of peptide bonds. Not only do peptidases hydrolyse proteins to amino acids and peptides for nutrition and recycling, but they also perform some of the most important post-translational processing events leading to the activation (or inactivation) of many other proteins, including other enzymes and peptide hormones. Over 2% of the protein coding genes in a genome encode peptidases, and there are over 500 peptidase genes in the human genome. Peptidases exist in at least six catalytic types, depending on the nature of the nucleophile in the catalytic reaction (either the hydroxyl of a serine or threonine, the thiol of a cysteine, or activated water bound either to a metal ion (metallopeptidases) or aspartate or glutamate residues). Peptidases are grouped into over 250 different evolutionary families [1].

The pepsin family (*MEROPS *family A1 [2]) contains aspartic-type endopeptidases. Activated water is bound by two aspartate residues each of which is located in a motif Xaa-Xaa-Asp-Xbb-Gly-Xbb where Xaa is a hydrophobic amino acid and Xbb is either Ser or Thr. A third important residue is a tyrosine which interacts with the substrate and is located on a beta-hairpin loop known as the "flap" which forms part of the roof to the active site. A second motif, part of a structure known as the psi-loop [3] consists of two hydrophobic residues followed by a glycine. Two of these motifs are conserved in all sequences known or predicted to be active within family A1. The psi-loops are important in stabilizing the structure, especially the active centre. Many members of family A1 have three conserved disulphide bridges. Homologues are known from animals, plants, fungi and protozoa, but no homologues have been reported from prokaryotes. Family A1 has recently been divided into two subfamilies, with the majority of characterized peptidases in subfamily A1A. Peptidases within this subfamily include: pepsin A (EC 3.4.23.1), gastricsin (EC 3.4.23.3) and chymosin (EC 3.4.23.4), which are digestive enzymes in the stomach; cathepsin D (EC 3.4.23.5), which is a lysosomal enzyme that degrades phagocytozed peptides and proteins; renin (EC 3.4.23.15), which is a peptidase that processes angiotensinogen; and memapsins 1 (or BACE-2, EC 3.4.23.45) and 2 (BACE-1, EC 3.4.23.46), widely distributed peptidases of unknown function but which may be involved in Alzheimer's disease (memapsin-2 is currently under investigation as a drug target [4]). Subfamily A1B contains predominantly peptidase homologues from plants including nucellin [5] and nepenthesin (EC 3.4.23.12; [6]). Besides peptidases, family A1 also contains a number of proteins which cannot be peptidases because the active site aspartates are not conserved. These include pregnancy-associated glycoproteins [7] and a xylanase inhibitor [8].

The tertiary structures of several members of this family have been determined, and each shows a bilobed protein. Each lobe contains one of the active site aspartates in a psi-loop and the two lobes are structurally similar. This similarity can only be observed in the tertiary structure because, apart from motifs contributing to each psi-loop, there is no observable similarity in the protein sequence. This led to the hypothesis that an ancestral gene duplication had taken place, followed by gene fusion [9,9]. This raises the question of how did the original gene product function, being only one half of a modern-day pepsin? The discovery of a peptidase, retropepsin (EC 3.4.23.16), within the genome of the human immunodeficiency virus (HIV) that also represents a single lobe of an extremely divergent pepsin (so divergent that it is the representative of a different family, A2, in *MEROPS*, but within the same clan or superfamily AA) and which has to dimerize to be active, provided evidence to answer this question [10]. Proteins with a single pepsin lobe also exist in eukaryotes. The structure of the *Saccharomyces cerevisiae *protein Ddi1 (DNA-damage inducible protein 1), which is not a peptidase, has been resolved and shown to contain a domain with a retropepsin-like structure, and the protein also has to dimerize to be active [11]. This domain was probably derived from a retroelement and represents an even more ancient divergence than that of the A1 and A2 families, as has been shown in a recent phylogeny of clan AA peptidases [12]. It has been assumed that the HIV peptidase represents the ancestral state and that the ancestral half-pepsin also had to dimerize to be active. However, it is also possible that the HIV peptidase was derived from a normal, bilobed pepsin when a virus captured half of its host's gene. This "chicken and egg" situation might be resolvable if pepsin homologues could be found in prokaryotes, because this would represent an extremely distant sequence divergence. If a prokaryote pepsin homologue contained only one lobe, then it would support the hypothesis of the ancient gene duplication and fusion event. However, if the prokaryote pepsin homologue were bilobed, then either a) this hypothesis is incorrect; b) the gene duplication precedes the most recent common ancestor of prokaryotes and eukaryotes; or c) the bacterial homologue genes were derived by horizontal gene transfer.

We report the discovery of the first prokaryote pepsin homologues in the completed genome sequences of several proteobacteria.

## Results and Discussion

Over 960 completely sequenced prokaryote genomes were analysed (914 bacterial and 55 archaean), and 953 of these contained no detectable homologue of pepsin. This included all of the archaean genomes. However, pepsin homologues were detected in seven bacterial genomes, and these are shown in Table 1. Characteristics of the predicted proteins of these homologues are shown in Table 2. All the bacteria containing pepsin homologues are members of the class Gammaproteobacteria, and all except *Marinomonas *are members of the order Alteromonadales; *Marinomonas *is a member of the order Oceanospirillales. Despite the genomes having been completely sequenced, no pepsin homologue was detected in the proteomes of the following *Shewanella *species: *S. baltica*, *S. frigidimarina*, *S. halifaxensis, S. oneidensis*, *S. pealeana*, *S. putrefaciens*, *S*. sp. ANA-3, *S*. sp. MR-4, *S*. sp. MR-7, *S*. sp. W3-18-1 or *S. woodyi*, nor in *Sinorhizobium meliloti*.

**Table 1:** Homologues of pepsin from completely sequenced bacterial genomes

***Species***	***Genome accession***	***ProteinID***	***Gene locus***	***Base pairs***
*Colwellia psychrerythraea*	CP000083	AAZ24813	CPS_0549	c(546351..547676)
*Marinomonas *sp. MWYL1	NC_009654	YP_001340503	Mmwyl1_1642	1847648..1848868
*Shewanella amazonensis*	CP000507	ABL98994	Sama_0787	c(969066..970403)
*Shewanella denitrificans*	CP000302	ABE54094	Sden_0804	923752..925041
*Shewanella loihica*	CP000606	ABO24862	Shew_2996	3556266..3557951
*Shewanella sediminis*	CP000821	ABV38171	Ssed_3567	4347419..4348963
*Sinorhizobium medicae*	CP000738	ABR61223	Smed_3492	2467002..2468108

Members of peptidase family A1 found in the proteomes derived from completely sequenced bacterial genomes. Base pairs in parentheses preceded by the letter "c" are on the complimentary strand with respect to the database entry.

**Table 2:** Predicted characteristics of bacterial homologues of pepsin

***Species***	***Length***	***Predicted active site residues***	***Alien_Hunter score (threshold)***
*Colwellia psychrerythraea*	441	D36, Y68, D300	6.975 (14.690)
*Marinomonas *sp. MWYL1	406	D49, F95, D274	13.692 (13.678)
*Shewanella amazonensis*	445	D37, Y69, D282	4.976 (13.455)
*Shewanella denitrificans*	429	D37, Y69, D287	8.983 (10.423)
*Shewanella loihica*	561	D41, Y73, D399	3.441 (15.338)
*Shewanella sediminis*	514	D41, Y88, D364	12.942 (15.906)
*Sinorhizobium medicae*	368	D53,F97,D243	5.649 8.762 (16.612)

Predicted active site residues are numbered according to the translation of the respective coding sequence. The threshold scores for Alien Hunter analysis are given in parenthesis after the raw score; a different threshold exists for each genome analysis. The pepsin homologue in *Sinorhizobium medica *is derived from a gene that is split between two 5000-residue windows in the Alien Hunter analysis, hence there are two scores.

BlastP searches of the NCBI non-redundant database using the bacterial pepsin homologues as queries returned only one other bacterial sequence. This was the A5A_A0203 gene product from *Vibrio cholerae *strain MZO-2 (UniProt accession A6A7Y6). This sequence consists of 382 residues with the peptidase unit predicted to be residues 14-257, and the active site residues to be Asp41, Phe87 and Asp249 (numbered according to the translated coding sequence). The genome of this particular strain is incomplete and no pepsin homologue is present in any of the other thirteen strains of *Vibrio cholerae *for which the genome sequences are complete. Because this sequence might be a contaminant, it has not been included in the analysis. A search of the UniProt sequence database using HMMER also failed to return any other sequences from bacteria. Two Psi-Blast searches of the NCBI non-redundant protein sequence database were undertaken using the amino acid sequences of the pepsin homologues from *Shewanella amazonesis *and *Marinomonas *as the seed sequences for 19 and 14 iterations respectively (returning 2888 known pepsin homologues); these also failed to find any new bacterial homologues.

Four homologues were found among the environmental samples nucleotide sequence database (see Table 3), and all were from marine environments. The predicted protein sequences of AACY023056044 and AACY024067159 were virtually identical. None of these fragments were identical to any known pepsin homologue, but each was most closely related to a homologue from *Shewanella *and are likely to be derived from at least three other bacterial species. Because these are fragments only, they are not considered further.

**Table 3:** Bacterial pepsin homologues in environmental samples

**Accession**	**source**	**closest pepsin homologue**	**% identity**
AACY023084992	Eastern Pacific ocean surface water	*Shewanella amazonensis*	38%
AACY023056044	Eastern Pacific ocean surface water	*Shewanella denitrificans*	31%
AACY024067159	Eastern Pacific ocean surface water	*Shewanella loihica*	43%
AAFZ01015571	microbial mat from gray whale carcass in the Santa Cruz Basin, Pacific Ocean (depth 1674 metres)	*Shewanella denitrificans*	37%

Several of the bacteria with pepsin homologues are marine and psychrophilic, including *Colwellia psychrerythraea *(formerly *Vibrio psychroerythus*) [13] and *Shewanella detrificans *[14]. *Shewanella loihica *is also marine but associated with deep-sea, hydrothermal vents. *Shewanella amazonensis *was isolated from marine sediments from the shallow waters of the Amazon river delta, and *Shewanella sediminis *from sediments in Halifax Harbour, Canada [15]. *Marinomonas *sp. MWYL1 is a salt marsh species initially isolated from the root surface of the grass *Spartina anglica *[16]. Exceptionally, *Sinorhizobium medicae *is found associated with root nodules of *Medicago *species [17]. There are completed genome for several other *Shewanella *species that are also marine (*S. baltica *and *S. frigidimarina*, for example), but these do not contain pepsin homologues, so there is no apparent correlation between environment and presence of a pepsin homologue.

**Figure 1:**
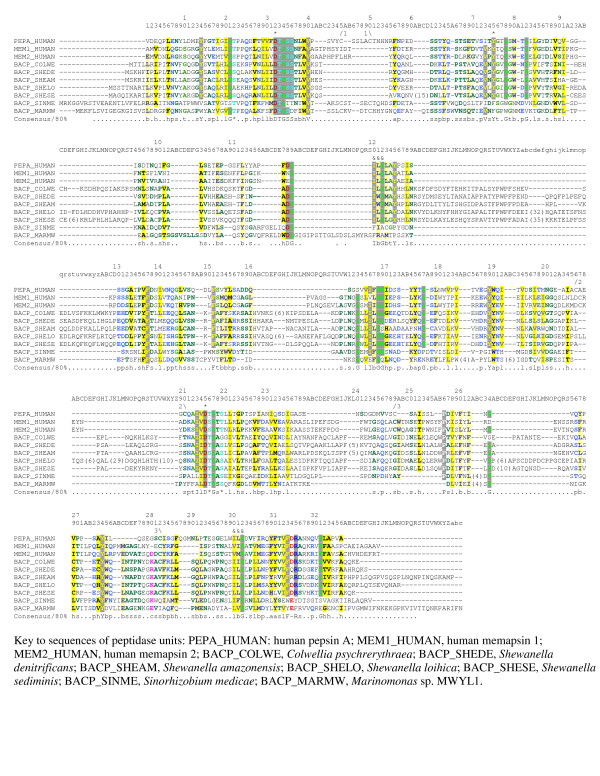
**Alignment of bacterial pepsin homologues with human pepsin A and memapsins 1 and 2**. Residues are numbered according to mature human pepsin A. Inserts relative to pepsin A are indicated by letters. Each active site residue (Asp32, Tyr75 and Asp215) is indicated by an asterisk. The hydrophobic-hydrophobic-Gly motifs in the psi-loops are indicated by ampersands. A disulphide bridge is indicated by a slash over one cysteine followed by a number and a backslash preceded by the same number over the second cysteine. The Chroma software [37] has been used to highlight residues according to amino acid properties and to generate the consensus line below each alignment block showing 80% conservation or more. Inserts found in only one sequence have been removed and are indicated by the number of amino acids excised in parentheses.

An alignment containing only bacterial homologues, human pepsin A and memapsins 1 and 2, generated by extracting the sequences from the MUSCLE alignment and removing all the gap-only columns, is shown in Fig. 1. As can be seen from this figure, both active site aspartates (Asp32 and Asp215) are conserved, indicating that the bacterial homologues are bilobed. This means that each homologue has the same structure for the peptidase unit as pepsin, and each would be active in the monomeric form.

None of the proteins were predicted to possess a signal peptide. This means that unlike the majority of members of peptidase family A1, these bacterial proteins are not secreted (the aspartic peptidase BcAP1 from the plant pathogenic fungus *Botrytis cinerea *also does not possess a signal peptide nor disulfide bonds [18]). Many members of family A1 are active at acidic pH, being secreted in the stomach, or to the lysosome or plant and fungal vacuoles, but a cytoplasmic peptidase would presumably be active at neutral pH. Amongst the mammalian pepsin homologues, renin is secreted into the blood and is also active at neutral pH. This change in pH optimum has been explained by the replacement of Thr218 by Ala, thereby preventing a hydrogen bond forming which affects the acidity of the active site residue Asp215. That this replacement is also found in the HIV retropepsin, which is also active at neutral pH, supports this hypothesis [19]. Intriguingly, six of the seven bacterial homologues also have Ala218 (see Fig. 1). In pepsin A, Thr218 is hydrogen-bonded to Asp303, but in renin both residues are replaced by Ala and site-directed mutagenesis of Ala303 for Asp lowered the pH optimum [20]. In the sequences from *Shewanella *and *Colwellia*, Asp303 is replaced by Leu, which is an isosteric replacement. In the *Sinorhizobium *sequence Asp303 is replaced by Arg, and in the *Marinomonas *sequence it is replaced by Ser. There would be no problem accommodating the smaller Ser in the available space, but the larger Arg could be more problematic.

Fig. 1 shows that of the cysteines forming the three disulphide bridges found in many of the eukaryotic homologues, only those forming the first are conserved in the *Sinorhizobium *and *Marinomonas *homologues. Cys282 from the third disulphide bridge is retained in the sequences from *Colwellia*, *Shewanella loihica *and *S. sediminis*, which may mean that the proteins are thiol-dependent. Disulphide bridges would not be expected in intracellular proteins. The *Sinorhizobium *and *Marinomonas *homologues also differ in having Phe75 instead of Tyr (although it must be acknowledged that the alignment here is by no means certain); although this is a common replacement, none of the homologues with Phe75 has ever been biochemically characterized or shown to be catalytically active. Replacement of Tyr75 for Phe in rhizopuspepsin by site-directed mutagenesis led to some, weakened activity [21]. The hydrophobic-hydrophobic-Gly motifs in the psi-loops are conserved in all the bacterial homologues except *Sinorhizobium *and *Marinomonas *. Because these bacterial homologues have inserts here, the alignment is uncertain. Although it is possible that the inserts might compensate for the loss of the hydrophobic-hydrophobic-Gly motif, which would imply a different structure in this region, it is much more likely that the two proteins lacking these motifs are not active as peptidases.

From the solved tertiary structure the residues forming the S1 and S1' substrate-binding pockets for human pepsin A are known. Pepsin A prefers large hydrophobic residues (Phe and Leu) in substrates for both P1 and P1', and the substrate-binding pockets are correspondingly lined with hydrophobic residues [22]. The bacterial homologues, with the exception of that from *Marinomonas*, have most of the substrate-binding residues conserved except Thr77, which is replaced by another hydrophobic residue, and Thr218, which is replaced by Ala (see Fig. 1).

**Figure 2:**
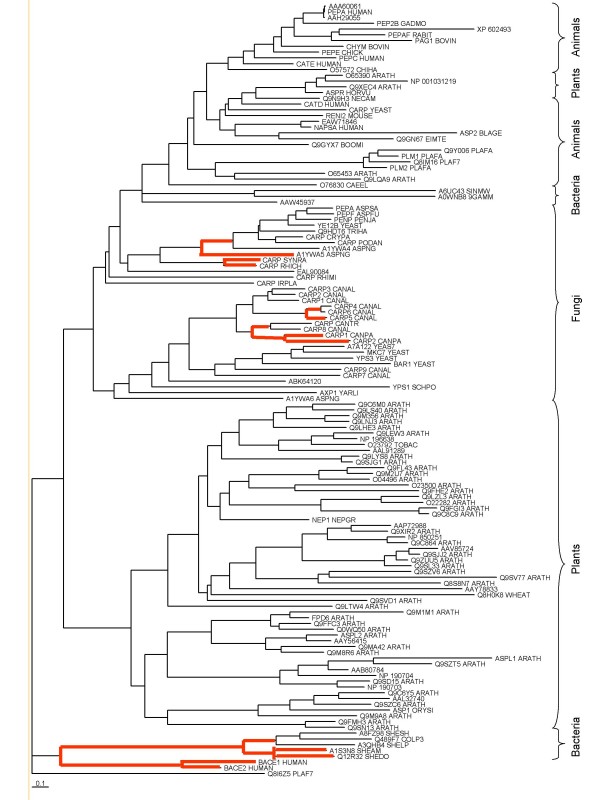
**Phylogenetic tree derived from members of peptidase family A1**. The tree was generated for all peptidase unit sequences of family A1 holotypes, plus those from the sequences of the bacteria listed in Table 1. The tree is unrooted, but the sequence of plasmepsin-5, which is very divergent, was chosen as the outgroup. Homologues from bacteria are highlighted with a blue background. Branches that were present in 90% of the bootstrap trees are shown in red. Key: [see Additional file 1].

The phylogenetic tree (Fig. 2) shows that all bar two of the bacterial sequences are close to the origin of the division between subfamilies on the tree, implying that horizontal transfer of genes from a recent eukaryote species to a bacterium is unlikely (the key is found in Additional File 1). This is confirmed by the results from Alien Hunter (see Table 2) which finds horizontal transfer of genes unlikely in all species except *Marinomonas*. Considering that *Marinomonas *is a commensal organism living on the root surface of a grass, the origin of a pepsin homologue via horizontal transfer of a gene from the host is not unexpected. The *Marinomonas *homologue is most closely related to that from *Sinorhizobium*. These sequences do not cluster with the other bacterial homologues on the tree. *Sinorhizobium *is also a commensal organism, living in the root nodules of legumes, but the region containing the gene was not predicted to be the result of horizontal transfer. There are therefore two groups of bacterial pepsins, one containing sequences from *Shewanella *and *Colwellia *and one containing sequences from *Sinorhizobium *and *Marinomonas*.

To further investigate whether the pepsin homologues in *Shewanella *species were derived from horizontal gene transfer, we attempted to estimate the rate of mutation. We hypothesized that if the bacterial pepsins are of ancient origin then their rate of mutation must be very low otherwise we would not be able to see the sequence similarity to eukaryotic proteins. We calculated the percentage identities between the pepsin sequences from *Shewanella *species and compared these with the percentage identities from other peptidase families. The families of signal peptidase 2 (peptidase family A8) and the ClpP subunit of endopeptidase Clp (family S16) were chosen because these are well conserved in bacteria, and all the *Shewanella *species have only one homologue per family. The percentage identities with respect to the homologues from *Shewanella denitrificans *are shown in Table 4, which shows that the ClpP subunit is the most stable, followed by signal peptidase 2 and then pepsin. The pepsin homologues are changing at twice the rate of the other two families. The closest homologues to *S. denitrificans *signal peptidase 2 and ClpP are the respective proteins from *S. loihica*. However, of the *Shewanella *pepsin homologues, that from *S. loihica *is most distantly related to *S. denitrificans*, even more distantly related than the sequence from a species in a different genus, *Colwellia*. To put the percentage identities into a geological time-frame, percentage identities were calculated for cathepsin D, a pepsin homologue found only in animals where divergence times can be estimated from the fossil record. The percentage identity between cathepsin D sequences from human and pig is 88% (human and pig diverged around 65 million years ago [23]), and human and *Xenopus tropicalis *is 73% (the species diverged around 350 million years ago [24]); while that of human cathepsin D and nemepsin-2 from the nematode *Ancylostoma caninum *is 52% (the species diverged around 660 million years ago [23]). The mutation rate for pepsin homologues amongst bacteria must therefore be considerably higher than eukaryote members of the family, and this rapid mutation rate might explain their placing near the root of the phylogenetic tree.

**Table 4:** Comparison of replacements among bacteria with pepsin homologues

**Family**	***Shewanella*** ***amazonensis***	***Shewanella*** ***loihica***	***Shewanella*** ***sediminis***	***Colwellia*** ***psychrerythraea***	***Sinorhizobium*** ***medicae***	***Marinomonas *sp.** **MWYL1**
A1 (pepsin)	55.5	42.4	43.6	41.3	17.1	15.5
A8 (signal peptidase 2)	70.8	72.4	67.6	48.3	35.8	46.4
S16 (endpeptidase Clp)	89.0	90.9	89.9	78.1	61.6	67.2

The protein sequences from *Shewanella denitrificans *were compared with orthologous protein sequences from other bacteria. Sequences were aligned with Muscle [30] and percentage identities calculated using Alistat .

The evidence as to whether the ancestral pepsin gene in bacteria originated from a horizontal gene transfer from a eukaryote or not can be summed up as follows. The positioning of most of the bacterial pepsins close to the divergence of the two subfamilies on the phylogenetic tree, the cytoplasmic location and lack of disulfide bridges and the scores from Alien Hunter imply no lateral transference; the absence of homologues in other closely related bacterial species and Archaea and the apparent fast mutation rate are points in favour of horizontal gene transfer.

If the bacterial genes are the result of lateral gene transference from an ancient eukaryote gene, then there is no known current eukaryote gene that resembles it, because nearly all modern pepsin homologues are secreted proteins and the ancient gene product would presumably be cytoplasmic. The eukaryotic peptidases that are most closely related to the bacterial homologues are the memapsins, as can be seen in Fig. 2. The memapsins and bacterial pepsins cluster with high confidence. It is possible that memapsins, which are widely distributed in mammalian tissues, might be closer to the ancestral peptidase from which all other vertebrate pepsin homologues are derived.

Aspartic-type peptidases unrelated to pepsin are known in bacteria, including the gpr peptidase from *Bacillus megaterium *(peptidase family A25) [25] and omptin from *Escherichia coli *(peptidase family A26) [26]. Neither peptidase is inhibited by pepstatin. Sporulation factor SpoIIGA (peptidase family U4) [27] has also been claimed to be an aspartic peptidase because of the presence of a single Asp-Ser-Gly motif (the protein is assumed to be active as a dimer), but this alone is not sufficient because a same motif occurs around the active site Asp of the serine-type peptidase subtilisin. Pepstatin-sensitive aspartic peptidases have previously been found in *Escherichia coli *and *Haemophilus influenzae*, but the sequences were not homologous to that of pepsin but homologues of gluconate permease [28]. A recent publication [29] has reported an acidic peptidase from a *Synergistes *species isolated from the anaerobic digester used for treatment of tannery solid waste. This peptidase is inhibited 75% by 0.01 mM pepstatin, but not by inhibitors of other catalytic types. No sequence is available, but this may be the first characterized pepsin homologue from a bacterium. Unlike the homologues from the marine bacteria, this is presumably a secreted protein. No complete genome sequence for any *Synergistes *species is publicly available, so we were unable to test for the presence of a pepsin homologue. The genomes of *Dethiosulfovibrio peptidovorans *and *Thermanaerovibrio acidaminovorans*, both of which are members of the same bacterial phylum Synergistetes, have been partially sequenced (genome projects 20741 and 29531, respectively). These were searched with the amino acid sequences of pepsin homologues from *Shewanella amazonensis *and *Sinorhizobium medicae*, but no pepsin homologues were found, even at an E value of 10.

## Conclusion

The pepsin family of peptidases (family A1) is not just confined to eukaryotes, but is also present in some bacteria. These bacterial homologues are predicted to be intracellular, whereas nearly all other members of family A1 enter the secretory pathway. The bacterial homologues are structurally similar to pepsin, consisting of two lobes each of which bears one active site Asp. Two of the genes for bacterial pepsins might be derived from horizontal gene transfer from a eukaryote, but whether all the bacterial genes for the bacterial pepsins are so derived is debatable, and evidence is presented for and against the case. If no horizontal gene transfer has occurred, then the hypothetical gene duplication and fusion event that gave rise to the ancestral pepsin gene would therefore have occurred before the divergence of bacteria and eukaryotes. If so, this has implications for the origin of the viral and retrotransposon peptidases in family A2, which were either derived from the single-lobed ancestral sequence, or secondarily from the horizontal transfer of half a pepsin gene from a host. For the retropepsin gene to be derived by vertical transfer the ancestral gene would have to have been present in a prokaryote. However, retroviruses infect only eukaryotes and retrotransposons are unknown in prokaryotes, making a more recent horizontal transfer the more likely origin of the retropepsin gene.

## Methods

### Data sources and analyses

All the complete, predicted proteomes (in FastA format) from bacterial genome sequencing projects were downloaded from the National Center for Biotechnology Information (NCBI) FTP site [30]. Each proteome was submitted to the *MEROPS *batch Blast Internet utility [31]. This service performs a BlastP search [32] against a representative set of peptidase sequences for every sequence in the proteome library. For each sequence that is detected as homologous to a peptidase sequence (where the expect value is less than 0.001) the extent of the peptidase unit is determined and the potential active site residues are predicted. The peptidase unit is defined as the subdomains determined from the tertiary structure of the type example peptidase (in this case human pepsin A) that bear the known active site residues.

The *MEROPS *batch Blast software determines to which peptidase family a sequence belongs. To further characterize the sequences, each sequence returned in the *MEROPS *batch Blast output was submitted to a further BlastP search against the complete library of peptidase unit sequences. During this second round of searching, homologous fragments are distinguished from possible false positive fragments by using a more rigorous expect value threshold (< e-10). A sequence was defined as a fragment if in the BlastP alignment between the query and hit sequences there was no overlap in the regions of sequence where active site residues were predicted to be.

Any pepsin homologue detected was used in a further BlastP search of the NCBI non-redundant protein sequence database to try to find more distantly related homologues. The protein sequence database at NCBI from environmental samples was also searched for homologues using BlastP. Additionally, TBlastN searches [32] were done against the environmental samples nucleotide sequence database and against all nucleotide sequences in the NCBI non-redundant nucleotide sequence database. The UniProt database was searched using the HMMER software  and a Hidden Markov Model built from any pepsin homologues found. Psi-Blast [32] searches of the NCBI non-redundant database were also undertaken, accumulating hits with an E-value of 0.005 or less.

### Sequence alignments

Sequences were aligned using MUSCLE [33] to holotypes of the pepsin family. In the *MEROPS *database a holotype sequence is chosen to be a representative of each different peptidase characterized biochemically. Holotypes have also been selected for every different human, mouse and *Arabidopsis thaliana *gene product predicted to be a peptidase homologue. Any sequences where the active site residues were not conserved were removed, leaving 135 sequences.

### Phylogenetic tree

A phylogenetic tree derived from the MUSCLE alignment was generated using the PROML (protein maximum likelihood) program from the Phylip package [34]. A consensus tree was also generated using the BOOTSEQ, PROML and CONSENSE programs from the Phylip package for a subset of the holotype library, retaining all holotypes from subfamily A1A plus the sequences of nepenthesin and nucellin from subfamily A1B. The number of bootstraps was 100, otherwise default values were used.

### Signal peptide prediction

The SignalP server  was used to predict signal peptides [35].

### Prediction of horizontally transfer DNA

In order to find regions where DNA might have been horizontally transferred between species, rather than vertically transferred from a common ancestor, the program Alien Hunter was used [36]. This program uses compositional biases (including CG content and third codon usage) to detect potential horizontally transferred genetic material. The default value window of 5000 bases was used. Any region scoring higher than the calculated threshold for the genome is potentially derived by horizontal transfer.

## Authors' contributions

NDR conceived the study, performed the sequence analyses and drafted the manuscript. AB participated in the design and helped to draft the manuscript. Both authors read and approved the final manuscript.

## Supplementary Material

Additional file 1 **Key to **Fig. 2. **(phylogenetic tree derived from members of peptidase family A1)**. the file contains the key to the tips of the phylogenetic tree. Click here for file
