# Supplementary material for: Pepsin homologues in bacteria
Source: BMC Genomics. 2009 Sep 16;10:437. doi: 10.1186/1471-2164-10-437 (PMC2761423; doi:10.1186/1471-2164-10-437)
Supplement: Additional file 1 — Key to Fig. 2. (phylogenetic tree derived from members of peptidase family A1). the file contains the key to the tips of the phylogenetic tree. [file 1471-2164-10-437-S1.doc]

Title: Key to Fig. 2. (phylogenetic tree derived from members of peptidase family A1)

Key: AAA60061, pepsin A5 (*Homo sapiens*); PEPA_HUMAN, pepsin A (*Homo sapiens*); AAH29055, pepsin A4 (*Homo sapiens*); PEP2B_GADMO, Mername-AA047 peptidase (*Gadus morhua*); XP_602493, pepsin homologue (*Bos taurus*); PEPAF_RABIT, pepsin F (*Oryctolagus cuniculus*); PAG1_BOVIN, pepsin F (*Bos taurus*); CHYM_BOVIN, chymosin (*Bos taurus*); PEPE_CHICK, embryonic pepsin (*Gallus gallus*); PEPC_HUMAN, gastricsin (*Homo sapiens*); CATE_HUMAN, cathepsin E (*Homo sapiens*); O57572_CHIHA, nothepsin (*Chionodraco hamatus*); O65390_ARATH, phytepsin (*Arabidopsis thaliana*); NP_001031219, At1g62290 (*Arabidopsis thaliana*); Q9XEC4_ARATH, At4g04460 (*Arabidopsis thaliana*); ASPR_HORVU, phytepsin (*Hordeum vulgare*); Q9N9H3_NECAM, nemepsin-2 (*Necator americanus*); CATD_HUMAN, cathepsin D (*Homo sapiens*); CARP_YEAST, saccharopepsin (*Saccharomyces cerevisiae*); RENI2_MOUSE, renin-2 (*Mus musculus*); EAW71846hCG1733572 putative peptidase (*Homo sapiens*); NAPSA_HUMAN, napsin A (*Homo sapiens*); ASP2_BLAGE, cockroach allergen (*Blattella germanica*); Q9GN67_EIMTE, eimepsin (*Eimeria tenella*); Q9GYX7_BOOMI, tick heme-binding aspartic proteinase (*Boophilus microplus*); Q9Y006_PLAFA, histoaspartic peptidase (*Plasmodium falciparum*); PLM1_PLAFA, plasmepsin-1 (*Plasmodium falciparum*); Q8IM16_PLAF7, plasmepsin-4 (*Plasmodium falciparum*); PLM2_PLAFA, plasmepsin-2 (*Plasmodium falciparum*); O65453_ARATH, At4g22050 (*Arabidopsis thaliana*); Q9LQA9_ARATH, At1g69100 (*Arabidopsis thaliana*); O76830_CAEEL, nemepsin-3 (*Caenorhabditis elegans*); A6UC43_SINMW, Smed_3492 protein (*Sinorhizobium medicae*); A0WNB8_9GAMM, Mmwyl1_1627 protein (*Marinomonas* sp. MWYL1); AAW45937, CnAP1 peptidase (*Cryptococcus neoformans*); PEPA_ASPSA, aspergillopepsin I (*Aspergillus saitoi*); PEPF_ASPFU, peptidase F (*Aspergillus fumigatus*); PENP_PENJA, penicillopepsin (*Penicillium janthinellum*); YE12B_YEAST, oryzepsin (*Aspergillus oryzae*); Q9HDT6_TRIHA, trichodermapepsin (*Trichoderma harzianum*); CARP_CRYPA, endothiapepsin (*Cryphonectria parasitica*); CARP_PODAN, podosporapepsin (*Podospora anserina*); A1YWA4_ASPNG, PepAa peptidase (*Aspergillus niger*); A1YWA5_ASPNG, PepAb peptidase (*Aspergillus niger*); CARP_SYNRA, syncephapepsin (*Syncephalastrum racemosum*); CARP_RHICH, rhizopuspepsin (*Rhizopus microsporus*); EAL90084, CtsD peptidase (*Aspergillus fumigatus*); CARP_RHIMI, mucorpepsin (*Rhizomucor miehei*); CARP_IRPLA, polyporopepsin (*Polyporus tulipiferae*); CARP3_CANAL, candidapepsin SAP3 (*Candida albicans*); CARP2_CANAL, candidapepsin SAP2 (*Candida albicans*); CARP1_CANAL, candidapepsin SAP1 (*Candida albicans*); CARP4_CANAL, candidapepsin SAP4 (*Candida albicans*); CARP6_CANAL, candidapepsin SAP6 (*Candida albicans*); CARP5_CANAL, candidapepsin SAP5 (*Candida albicans*); CARP_CANTR, canditropsin (*Candida tropicalis*); CARP8_CANAL, candidapepsin SAP8 (*Candida albicans*); CARP1_CANPA, candiparapsin (*Candida parapsilosis*); CARP2_CANPA, Sapp2p peptidase (*Candida parapsilosis*); A7A122_YEAS7, yapsin-1 (*Saccharomyces cerevisiae*); MKC7_YEAST, yapsin-2 (*Saccharomyces cerevisiae*); YPS3_YEAST, yapsin-3 (*Saccharomyces cerevisiae*); BAR1_YEAST, barrierpepsin (*Saccharomyces cerevisiae*); CARP9_CANAL, candidapepsin SAP9 (*Candida albicans*); CARP7_CANAL, candidapepsin SAP7 (*Candida albicans*); ABK64120, SA76 peptidase (*Trichoderma harzianum*); YPS1_SCHPO, Yps1 protein (*Schizosaccharomyces pombe*); AXP1_YARLI, axp peptidase (*Yarrowia lipolytica*); A1YWA6_ASPNG, PepAc peptidase (*Aspergillus niger*); Q9C6M0_ARATH, At1g25510 (*Arabidopsis thaliana*); Q9LS40_ARATH, At3g18490 (*Arabidopsis thaliana*); Q9M356_ARATH, At3g61820 (*Arabidopsis thaliana*); Q9LNJ3_ARATH, At1g01300 (*Arabidopsis thaliana*); Q9LHE3_ARATH, At3g20015 (*Arabidopsis thaliana*); Q9LEW3_ARATH, At5g10760 (*Arabidopsis thaliana*); NP_196638, At5g10770 (*Arabidopsis thaliana*); O23792_TOBAC, CND41 peptidase (*Nicotiana tabacum*); AAL91289, At1g79720 (*Arabidopsis thaliana*); Q9LYS8_ARATH, At3g59080 (*Arabidopsis thaliana*); Q9SJG1_ARATH, At2g42980 (*Arabidopsis thaliana*); Q9FL43_ARATH, At5g07030 (*Arabidopsis thaliana*); Q9M2U7_ARATH, At3g54400 (*Arabidopsis thaliana*); O04496_ARATH, At1g09750 (*Arabidopsis thaliana*); O23500_ARATH, At4g16560 (*Arabidopsis thaliana*); Q9FHE2_ARATH, At5g45120 (*Arabidopsis thaliana*); Q9LZL3_ARATH, PCS1 peptidase (*Arabidopsis thaliana*); O22282_ARATH, At2g39710 (*Arabidopsis thaliana*); Q9FGI3_ARATH, At5g37540 (*Arabidopsis thaliana*); Q9C8C9_ARATH, At1g66180 (*Arabidopsis thaliana*); NEP1_NEPGR, nepenthesin (*Nepenthes gracilis*); AAP72988, CDR1 peptidase (*Arabidopsis thaliana*); Q9XIR2_ARATH, At1g64830 (*Arabidopsis thaliana*); NP_850251, At2g35615 (*Arabidopsis thaliana*); Q9C864_ARATH, At1g31450 (*Arabidopsis thaliana*); AAV85724, At2g28040 (*Arabidopsis thaliana*); Q9SJJ2_ARATH, At2g28010 (*Arabidopsis thaliana*); Q9ZUU5_ARATH, At2g28030 (*Arabidopsis thaliana*); Q9SL33_ARATH, At2g28220 (*Arabidopsis thaliana*); Q9SZV6_ARATH, At4g30030 (*Arabidopsis thaliana*); Q9SV77_ARATH, At4g12920 (*Arabidopsis thaliana*); Q8S8N7_ARATH, At2g23945 (*Arabidopsis thaliana*); AAY78833, At5g24820 (*Arabidopsis thaliana*); Q8S8N7_ARATH, At2g23945 (*Arabidopsis thaliana*); Q8H0K8_WHEAT, xylanase inhibitor precursor (*Triticum aestivum*); Q9SVD1_ARATH, At3g52500 (*Arabidopsis thaliana*); Q9LTW4_ARATH, At3g12700 (*Arabidopsis thaliana*); Q9M1M1_ARATH, At3g42550 (*Arabidopsis thaliana*); FPD6_ARATH, **XXXXXX** (*Arabidopsis thaliana*); Q9FFC3_ARATH, At5g22850 (*Arabidopsis thaliana*); Q0WQ50_ARATH, At2g36670 (*Arabidopsis thaliana*); ASPL2_ARATH, At1g65240 (*Arabidopsis thaliana*); AAY56415, At5g36260 (*Arabidopsis thaliana*); Q9MA42_ARATH, At1g05840 (*Arabidopsis thaliana*); Q9M8R6_ARATH, At3g02740 (*Arabidopsis thaliana*); ASPL1_ARATH, At5g10080 (*Arabidopsis thaliana*); Q9SZT5_ARATH, At4g35880 (*Arabidopsis thaliana*); AAB80784, At2g17760 (*Arabidopsis thaliana*); NP_190704, At3g51360 (*Arabidopsis thaliana*); Q9SD15_ARATH, At3g51340 (*Arabidopsis thaliana*); NP_190703, At3g51350 (*Arabidopsis thaliana*); Q9C6Y5_ARATH, At1g44130 (*Arabidopsis thaliana*); AAL32740, At1g77480 (*Arabidopsis thaliana*); Q9SZC6_ARATH, At4g33490 (*Arabidopsis thaliana*); ASP1_ORYSI, nucellin (*Oryza sativa*); Q9M9A8_ARATH, At1g49050 (*Arabidopsis thaliana*); Q9FMH3_ARATH, At5g43100 (*Arabidopsis thaliana*); Q9SN13_ARATH, At3g50050 (*Arabidopsis thaliana*); A8FZ98_SHESH, Shew_2996 protein (*Shewanella sediminis*); Q489F7_COLP3, CPS_0549 protein (*Colwellia psychrerythraea*); A3QHB4_SHELP, Ssed_3567 protein (*Shewanella loihica*); A1S3N8_SHEAM, Sama_0787 protein (*Shewanella amazonensis*); Q12R32_SHEDO, Sden_0804 protein (*Shewanella denitrificans*); BACE1_HUMAN, memapsin-2 (*Homo sapiens*); BACE2_HUMAN, memapsin-1 (*Homo sapiens*); Q8I6Z5_PLAF7, plasmepsin-5 (*Plasmodium falciparum*).
